# Immunopotentiators Improve the Efficacy of Oil-Emulsion-Inactivated Avian Influenza Vaccine in Chickens, Ducks and Geese

**DOI:** 10.1371/journal.pone.0156573

**Published:** 2016-05-27

**Authors:** Jihu Lu, Peipei Wu, Xuehua Zhang, Lei Feng, Bin Dong, Xuan Chu, Xiufan Liu, Daxin Peng, Yuan Liu, Huailiang Ma, Jibo Hou, Yinghua Tang

**Affiliations:** 1 National Research Center of Engineering and Technology for Veterinary Biologicals, Ministry of Agriculture, Key Laboratory of Veterinary Biological Engineering and Technology, Jiangsu Academy of Agricultural Sciences, Nanjing, Jiangsu, PR China; 2 College of Veterinary Medicine, Yangzhou University, Jiangsu Co-Innovation Center for the Prevention and Control of Important Animal Infectious Disease and Zoonoses, Yangzhou, Jiangsu, PR China; 3 Program of Cellular Biology and Immunology, Department of Biology, Center for Inflammation, Immunity and Infection, Georgia State University, Atlanta, Georgia, United States of America; 4 Emory Vaccine Center, Yerkes National Primate Research Center, Emory University, Atlanta, Georgia, United States of America; Sun Yat-sen University, CHINA

## Abstract

Combination of CVCVA5 adjuvant and commercial avian influenza (AI) vaccine has been previously demonstrated to provide good protection against different AI viruses in chickens. In this study, we further investigated the protective immunity of CVCVA5-adjuvanted oil-emulsion inactivated AI vaccine in chickens, ducks and geese. Compared to the commercial H5 inactivated vaccine, the H5-CVCVA5 vaccine induced significantly higher titers of hemaglutinin inhibitory antibodies in three lines of broiler chickens and ducks, elongated the antibody persistence periods in geese, elevated the levels of cross serum neutralization antibody against different clade and subclade H5 AI viruses in chicken embryos. High levels of mucosal antibody were detected in chickens injected with the H5 or H9-CVCA5 vaccine. Furthermore, cellular immune response was markedly improved in terms of increasing the serum levels of cytokine interferon-γ and interleukine 4, promoting proliferation of splenocytes and upregulating cytotoxicity activity in both H5- and H9-CVCVA5 vaccinated chickens. Together, these results provide evidence that AI vaccines supplemented with CVCVA5 adjuvant is a promising approach for overcoming the limitation of vaccine strain specificity of protection.

## Introduction

Avian influenza viruses (AIVs) not only lead to massive economic loss in poultry industry but also cause dangerous issue to human public health. The highly pathogenic H5N1 AIVs have evolved into more than ten distinct phylogenetic clades based on their hemagglutinin (HA) genes [1], and more than five genotypes of H9N2 influenza viruses have been detected [2–4]. Nationwide routine vaccination programs are utilized as part of a wide range of strategies to prevent and control influenza disease spread in the poultry industry in five countries or districts [5, 6]. However, the genetic mutations allow influenza virus easily to evade from the vaccine induced protective immunity.

The inactivated avian influenza vaccine is not able to provide a robust protection of cross-reactive and mucosal antibodies against the circulating mutant viruses in the field [7]. To date, nine H5 subtype [8] and at least fifteen H9 subtype virus seed strains have been produced and used for inactivated vaccine in China by matching the immunogenicity of the predominant circulating influenza viruses. However, the procedures of selection and development new vaccine candidates are time- and labor-consuming efforts. New vaccine candidates that do not exactly match to the next predominant circulating viruses also occurred occasionally. Aquatic birds, including the domestic ducks and geese, are considered as the reservoir and “silent” spread of AI viruses to chickens and other poultry [9]. The immune response to H5 subtype inactivated vaccine in the ducks or geese are not as good as in the chickens [10], and two-injection regimen is required to elicit a robust protection. Hence, improving the efficacy of the current available commercial vaccine is necessary in field applications of aquatic birds.

Adjuvant has been licensed in human influenza vaccine, papillomavirus vaccine and hepatitis B virus vaccine [11, 12]. The agonists of the pattern recognition receptors are the critical activators of host innate immunity [13, 14]. In particular, those agonists are reported to modulate antibody and T helper lymphocyte responses, which added in some inactivated virus-based vaccines as one of the vaccine components [15, 16]. We previously reported that the adjuvant, CVCVA5, can significantly improve the protection of commercial H5 and H9 inactivated vaccine in chickens [15]. In this study, we tested the efficacy of adjuvant CVCVA5 with H5 and H9 vaccines in broiler chickens, ducks and geese. We also evaluated the efficacy of adjuvant CVCVA5 with H5 and H9 vaccines in improving the production of cross-neutralization and mucosal antibodies in chickens. In addition, serum levels of IFN-γ and IL-4, splenocytes proliferation and cytotoxic lymphocyte (CTL) immune responses were investigated.

## Materials and Methods

### Ethics Statement

All animal studies were carried out in strict accordance with the recommendations in the National Guide for the Care and Use of Laboratory Animals. The protocol was approved by the Review Board of National Research Center of Engineering and Technology for Veterinary Biologicals, Jiangsu Academy of Agricultural Sciences. The surgery and euthanasia was performed under anesthesia with sodium pentobarbital solution (100 mg/kg body weight) via intravenous route to minimize suffering.

### Vaccines and viruses

The H5 vaccine (Weike Biotechnology Co., Harbin, China, Lot. No:20120326) and the corresponding antigen (H5-Re5, Weike, Lot. No:2012004) in heamagglutinin inhibition (HI) assay are commercially available. The H9 subtype AI vaccine (A/Chicken/NJ/02/2001, NJ02/01) was prepared as a previously described in a water-in-oil form [15]. Briefly, the H9 subtype virus strain NJ02/01 was propagated in allantoic cavities from 10- to 11-day-old specific pathogen free (SPF) embryonated chicken eggs. The viral allantoic fluids (EID_50_, 10^8.0^/0.1 ml) were purified by centrifugation (28,000×g, 30 min, 4°C) and inactivated with beta-propiolactone (v/v 0.5%, 24 h, 37°C, Sigma, St. Louis, MO). The purified virus recovered in the same volume of phosphate buffer solution (pH 7.2, PBS) buffer was added into Marcol 52 mineral oil (ESSO, Paris, France) to produce a water-in-oil emulsion vaccine (v/v, 1:3). H5 subtype viruses of A/Mallard/Huadong/S/2005 (S, H5N1, clade 2.3.4), A/Chicken/Zhejiang/2011 (ZJ, H5N2, clade 2.3.4.6) and A/Chicken/Huadong/4/2008 (DT, H5N1, clade 7) [17] were provided by Prof. Daxin Peng (Yangzhou University). All experiments involved in the live H5 subtype viruses were performed in the biosafety level 3 laboratory facilities.

### Adjuvants

The recipe of adjuvants, CVCVA5, was described in previous reports with two different use forms [15]. In one of the use patterns, the adjuvants, CVCVA5, were used as the vaccine chaperons, such as the adjuvanted H5 commercial vaccine (H5-CVCVA5). The preparation process of adjuvant was similar to the procedure of inactivated H5 vaccine in a water-in-oil emulsion. Briefly, the adjuvant components of poly I:C (InvivoGen, San Diego, CA), MDP in L–D isoform (InvivoGen) and levamisole hydrochloride (Sigma) were dissolved in PBS as aqueous phase. Additional adjuvant components resiquimod (InvivoGen) and imiquimod (InvivoGen) were dissolved in Marcol 52 mineral oil (ESSO) as oil phase. One volume aqueous phase adjuvant was mixed with three volume oil phase adjuvant. The water-in-oil emulsion form of CVCVA5 adjuvant mixed with the commercial H5 vaccine before injection. In another use form, the components of the adjuvants were added in the aqueous phase or oil phase, respectively, during the inactivated vaccine preparation process, such as the adjuvanted H9 inactivated vaccine (H9-CVCVA5). Both forms of adjuvants were preserved at 4°C for a period of 15-month, and their shelf-life stability was tested in a groups of 14-day-old specific pathogen free (SPF) white Leghorn chickens (n = 10) with total dose of 0.5 ml in volume.

### Adjuvant efficacy in broiler chickens, ducks and geese

The efficacy of the adjuvant CVCVA5 on H5 (Re-5) vaccine was evaluated in three broilers breeds (*G*. *gallus domesticus*), including the white feather broilers, yellow feather broilers and dot feather broilers, as well as aquatic poultry, the Mallard duck (*Anas platyrhynchos*) and domestic geese (*Anser cygnoides*). Groups of twenty 10- to 15-day-old chickens (Pukou Poultry, Jiangsu, China) were adopted to test the efficacy of adjuvants on H5 vaccine in each broilers breed that received a single dose of H5 or H5-CVCVA5 vaccines via subcutaneous route with volume of 0.5 ml, respectively. The sera were collected at 2-, 3- and 4-week post-vaccination (wpv). Both ducks and geese were obtained from Shengjia Poultry, Jiangsu, China. The maternal antibodies of hemagglutinn inhibition (HI) against H5 (Re-5) were less than 2 log_2_ before vaccination. Groups of ten 14-day-old mallard ducks received only a single dose of vaccines via subcutaneous route injection (0.5 ml) of H5 or H5-CVCVA5 vaccines, and naïve control, respectively. All birds were bled on week 2, 3 and 4 post-vaccination for serum collection. The efficacy of the two-shot vaccination was assessed in the domestic geese. Briefly, three groups of twenty 14-day-old goslings were received the prime vaccination subcutaneously of H5 or H5-CVCVA5 vaccines (0.5 ml), respectively, and a third group was set as non-immunized control group. Four weeks after prime vaccination, geese in each group were boosted with the same dose of vaccine as used in the prime injection via subcutaneous route. The geese were bled at 3-, 4- and 8-week, and then at 4-week interval thereafter, until 32 wpv. The serum antibody titers were measured by HI assay.

### Serum cytokine and mucosal antibody test

The T-helper (Th) type-1 cytokine IFN-γ (Invitrogen, CA, Lot. No:131401) and Th2-type cytokine IL-4 (USCN, Wuhan, China, Lot. No: E18030353) in chicken serum were detected by commercially available ELISA kit following the manufacturer’s instructions. Briefly, four stock serum samples from SPF chickens of each group were measured by ELISA Kit at three-week post vaccination from each group.

The mucosal antibody from the tracheal, bronchoalveolar lavage [18] fluids and small intestine mucus were detected by HI test. Briefly, the BAL fluids were prepared prior to obtain the tracheal mucus. Twenty-milliliter syringe that prefilled with 10 ml of phosphate buffer solution (PBS, pH 7.2) was instilled into the bronchus and lung, and suctioned back to syringe. This procedure was repeated for 10 times, and 1 ml BAL fluid was retrieved for antibody titer measurement. The sections of tracheal tissues from larynx to the junction of trachea and bronchus were employed to scrape tracheal mucus, and washed with 0.5 ml PBS. Ten-centimeter long sections of small intestine following the bile duct were used for intestine mucus collection. After clearing the intestine contents, the mucus was collected by gently scraping with a glass slide and transferred into 2 ml ice-cold PBS. The scraps and fluids were centrifuged at 10,000×g at 4°C for 15 min to remove the particulate materials. The supernatant containing the crude intestinal mucus were applied for HI test.

### Serum neutralization assay

SN assays were performed with alpha method. All serum samples from chickens were heat inactivated (56°C, 30 min). The antisera stock solutions were mixed with equal volumes of tenfold serial dilutions of H5 subtype variant virus solutions. The variant strains were included viruses from subclade 2.3.4.6 (A/Chicken/ Zhejiang/2011, ZJ, H5N2) and clade 7 (A/Chicken/Huadong/4/2008, DT, H5N1). The control viruses were from clade 2.3.4 (A/Mallard/Huadong/S/2005, S, H5N1), a homologous strain to the Re-5 vaccine virus. After 1 hour incubation at 37°C, the mixtures were inoculated into 11-day old SPF chicken embryos and the embryos were incubated and observed daily for up to 5 days. The death embryos or embryos with positive HA titer were used to determine the end-point titers that were calculated as the reciprocal of sera to neutralize the highest viruses contents in 50% of the eggs.

### Lymphocyte proliferation

The study was carried out about the lymphocyte proliferation response of the splenocytes which derived from SPF chickens administered by the H5 subtype vaccine (Re-5) or the H9 subtype vaccine (NJ02/01) with or without CVCVA5 adjuvant to the inactivated H5 or H9 antigen, respectively. The inactivated H5 subtype viral antigen (Re-5) was purified from the H5 HI test antigen, and the inactivated H9 subtype viral antigen (NJ02/01) was purified from inoculated SPF chicken embryo allantoic fluids. At 3 days post immunization, single cell suspension was generated from the harvested spleens in PBS (pH 7.2) that supplemented with 1% penicillin/streptomycin. The splenocytes were separated with a chicken lymphocyte separation medium (HaoYang Co., Nankai, China), pelleted at 1000 rpm for 10 min, resuspended in RPMI 1640 medium supplemented with 10% chicken serum and 1% penicillin / streptomycin. Viable splenocytes were added to 96-well plates in 0.1 ml at 1×10^6^ cells/well and incubated in triplicate with H5 (Re-5) or H9 (NJ02/01) inactivated viral antigen (5μg/ml), mitogen phytohemagglutinin with the concentrations of 25μg/ml (Sigma, China) was used as the positive control, or medium alone as the negative control, followed by 68 h incubation at 37°C in an atmosphere of 5% CO2. The lymphocyte proliferation response was evaluated by MTT (3-[4,5-dimethylthiazol-2-yl]-2,5 diphenyl tetrazolium bromide) assay with cell proliferation assay kit. Data was reported as stimulation index (SI), which was the mean of experimental wells/mean of antigen free wells (negative control).

### Cytotoxic T lymphocytes (CTL) assay

CTL activity was measured by the non-radioactive alternative method of lactate dehydrogenase (LDH) cytotoxicity assay (Takara, Kyoto, Japan, Lot. No: AE5P027), which detects the stable cytosolic enzyme LDH released from lysed cells. The assay was performed according to manufacturer’s instruction and the previous study [19]. Briefly, the target cells that derived from the inbred SPF chickens (B_19_/B_19_) embryo fibroblast cells were infected for 8 hours with S (H5N1) virus with multiplicity of infection of 1, or 10 hours with NJ02/01 (H9N2) virus at multiplicity of infection of 2. The effector cells derived from peripheral blood mononuclear cells isolated from the inbred SPF chickens at 21 days post-vaccination, which previously immunized with the H5 subtype vaccine (Re-5) or the H9 subtype vaccine (NJ02/01) with or without CVCVA5 adjuvants, respectively. Various amounts of effector T cells in 100 μl of RPMI 1640 supplemented with 10% chicken serum, 1% L-glutamine, 1% sodium pyruvate, and 1% MEM nonessential amino acids were added to each well. 10^4^ target cells (100 μl /well) infected or uninfected were also seeded to each well. Each cell sample was plated in triplicate. Microtiter plates were centrifuged at 250×g for 5 min before they were incubated for 4 hours in a humidified chamber at 37°C, 5% CO_2_. After 4 hours, the plates were centrifuged, the supernatant was harvested, and the substrate tetrazolium salt was added. The OD values were read at 490 nm in an enzyme-linked immunosorbent assay reader. The specific LDH activity release was calculated by using the following formula: (experimental release-spontaneous release)/(maximum release -spontaneous release) ×100.

## Results

### Adjuvant H5-vaccine improves antibody response without side-effect on production performance in broiler chickens

**Fig 1:**
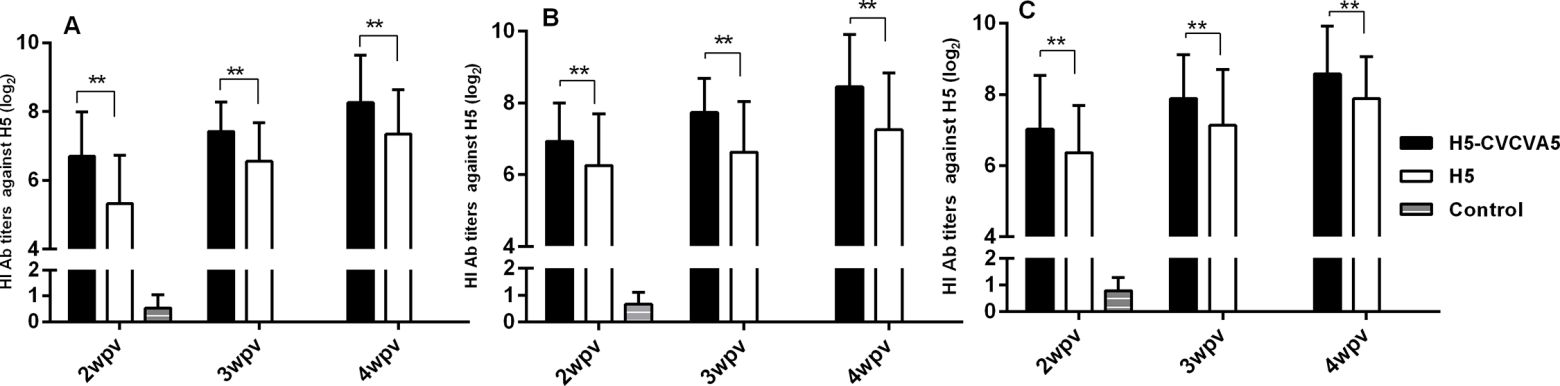
The HI antibody titers against H5 viral antigen in broilers. White feather broilers (A), yellow feather broilers (B), or dot feather broilers (C). The chickens (n = 20) vaccinated at 10–15 days old, and serum collected at 2-, 3-, and 4-week post-vaccination (wpv). H5-CVCVA5, commercial vaccine (H5-Re5) mixed with CVCVA5 adjuvants. H5, commercial vaccine (H5-Re5). Control, naïve control group. **, *P* < 0.05. Error bars indicates SEM.

Three lines of broilers with different growth period were employed to evaluate the efficacy of the adjuvants, CVCVA5, on improving the antibody response levels elicited by the H5 subtype inactivated vaccine. Compared to those of chickens injected with commercial H5-vaccine without adjuvant, the adjuvant H5-vaccine immunized white feather broilers demonstrated a significantly higher HI antibodies levels throughout the 4 weeks course of vaccination (at least 0.7 log_2_, data not shown) (Fig 1A). The chickens received H5-CVCVA5 vaccine were one week ahead of the chickens received commercial H5-vaccine to achieve the qualified anti-H5 virus antibody titer (6 log_2_). The similar effects of CVCVA5 adjuvant on improving of HI antibodies level elicited by H5 vaccine were also observed in yellow or dot feather broilers, respectively (Fig 1B and 1C).

The impact of adjuvant on the production performance of three species broilers was further assessed according to three indexes, including the slaughter weight, the ratio of feed conversion, and the ratio of death and culling after vaccination. The H5-CVCVA5 vaccinated white feather broilers showed no obvious difference based on the three indexes to those birds which immunized with the commercial vaccine or the unvaccinated control group (Table 1). Similar results were detected in the other two breeds of broilers. Collectively, these data indicated that addition of the adjuvant CVCVA5 to the H5 vaccine did not affect the broilers production performance.

**Table 1:** Broilers production performance.

Broiler Species	Vaccine	Day of slaughter	Slaughter weight (g)	Feed conversion ratio	Death and culling ratio
White feather	H5-CVCVA5	42	1864	2.24	0
White feather	H5	42	1853	2.25	0
White feather	Control	42	1870	2.18	0
Yellow feather	H5-CVCVA5	68	1754	2.36	0
Yellow feather	H5	68	1742	2.48	0
Yellow feather	Control	68	1743	2.37	0
Dot feather	H5-CVCVA5	110	1785	3.27	0
Dot feather	H5	110	1798	3.31	0
Dot feather	Control	110	1767	3.36	0

### Adjuvant H5-vaccine induces higher antibody response in ducks and geese

**Fig 2:**
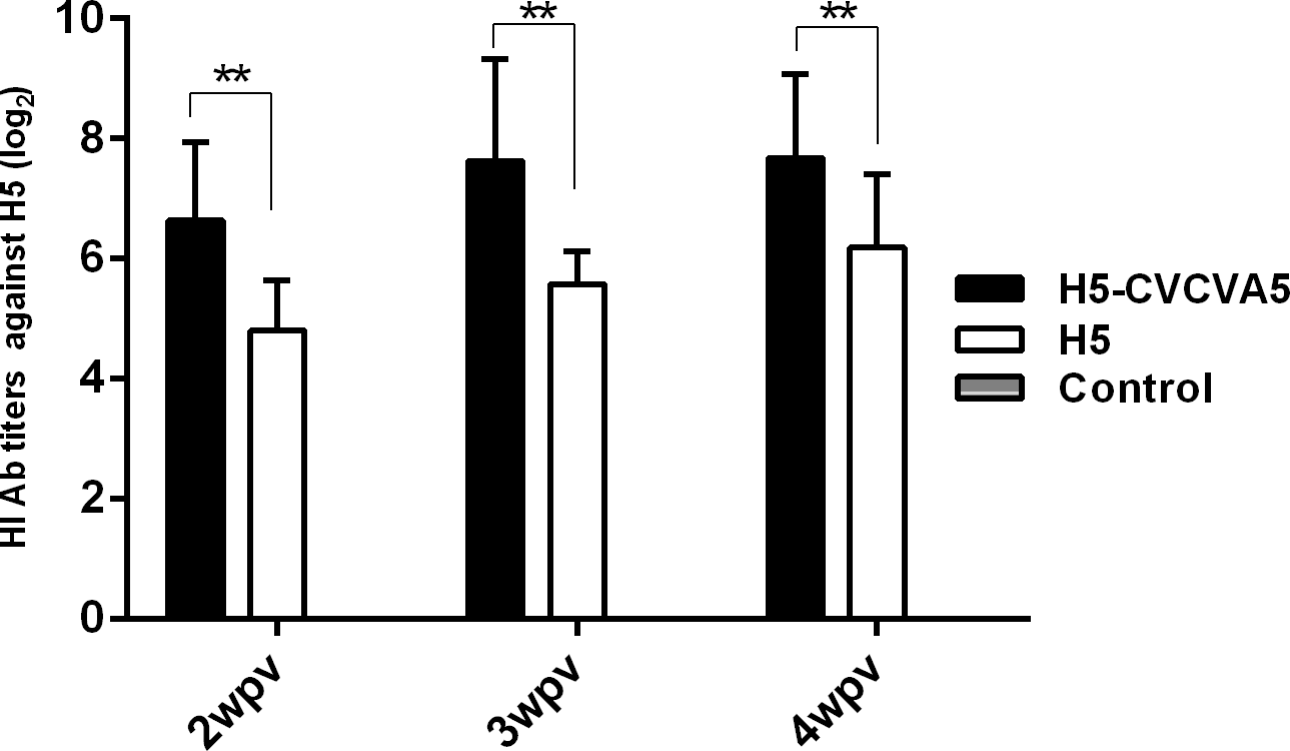
The HI antibody titers against H5 viral antigens in Mallard duck. The ducks (n = 10) vaccinated at 14-day old, and serum were collected at 2–3-, and 4-week post-vaccination (wpv). H5-CVCVA5, commercial vaccine (H5-Re5) mixed with CVCVA5 adjuvants. H5, commercial vaccine (H5-Re5). Control, naïve control group. **, *P* < 0.05.

**Fig 3:**
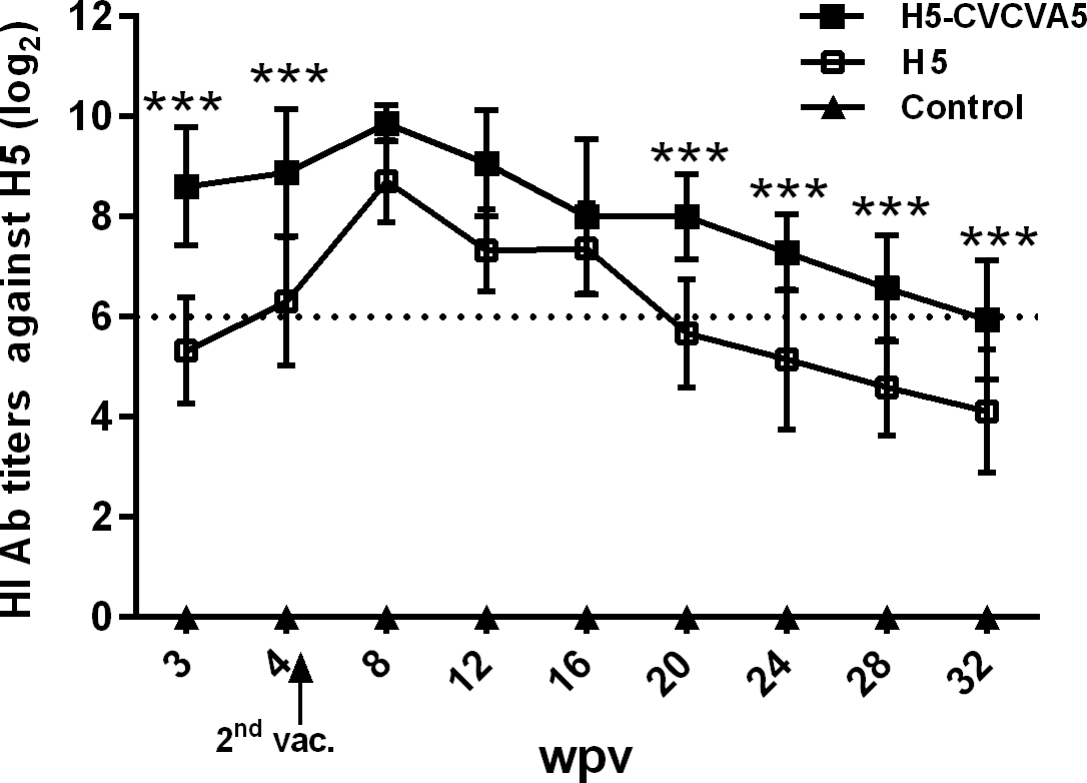
The HI antibody titers against H5 viral antigen in domestic geese. Groups of twenty 14-day-old goslings via subcutaneously route received the prime-boost vaccination of H5 or H5-CVCVA5 vaccines (0.5ml), respectively, and the third group was set as blank control. 2^nd^ vac., the second vaccination. The horizontal dash line is the qualified antibody titer according to the requirements of commercial vaccine. ***, *P* < 0.01.

The HI antibody titers of the H5-CVCVA5 vaccinated ducks were significantly higher than the birds which only injected with the H5 vaccine during the detection period from 2- to 4-week post-vaccination (Fig 2). The immune persistence periods of qualified antibody level, 6log_2_, were elongated to approximately 12 weeks in the boosted geese (Fig 3). The levels of antibodies specific for H5 viral antigen elicited by the vaccine of H5-CVCVA5 in geese were higher than those of the commercial H5 vaccine group over the 32-week monitoring period. The HI serum titers of the geese in the H5 commercial vaccine group were rapidly decline to unqualified antibody titer at 20-week post-vaccination. In contrast, the HI antibody titers of geese in H5-CVCVA5 vaccine group were maintained high levels and persist to 32-week post-vaccination.

### Long shelf-life of CVCVA5 adjuvants

**Fig 4:**
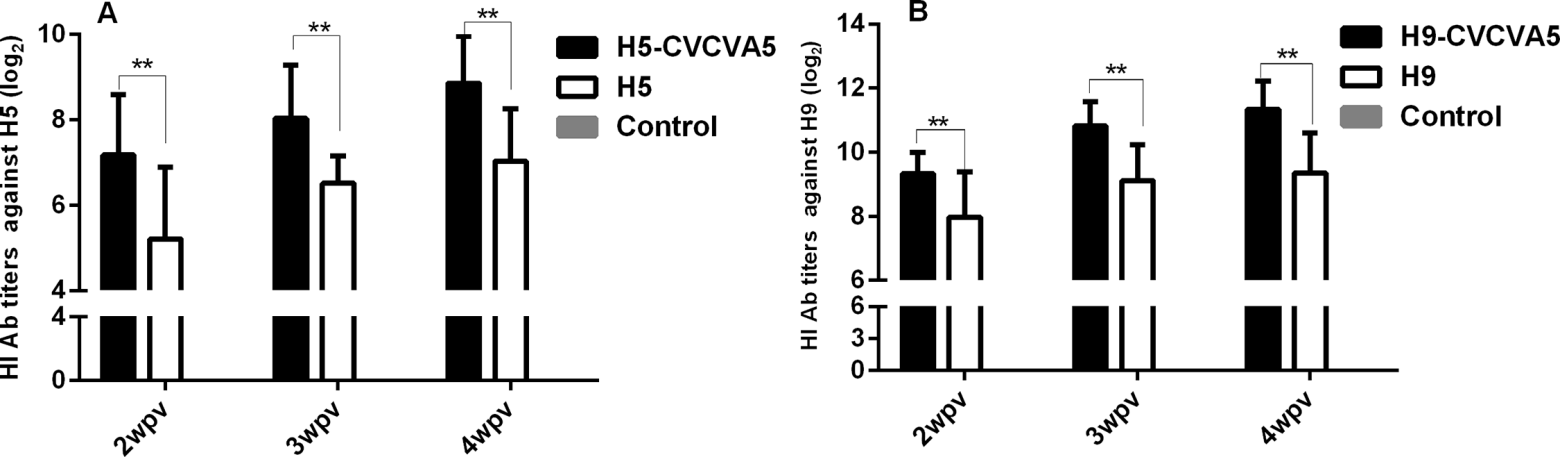
The HI antibody titers against H5 or H9 viral antigen in shelf-life test of CVCVA5. The adjuvants in H5-CVCVA5 were prepared in exclusive water-in-oil form which stored at 4°C for 15-month period, and mixed with the H5 (Re-5) commercial inactivated vaccine (A). The H9-CVCVA5 was prepared as all components of the adjuvants directly addition to the aqueous or oil phase of H9 vaccine during the preparation processes, and preserved for a period of 15-month at 4°C (B). **, *P* < 0.05.

The efficacy of adjuvants CVCVA5 after been stored at 4°C for a period of 15-month was tested with H5 or H9 subtype inactivated vaccine in SPF chickens. As a chaperon form of immune adjuvants, the CVCVA5 adjuvant H5 vaccine considerably improved by the HI serum antibody levels at 2-, 3- and 4-wpv when compared to the H5 commercial inactivated vaccine without adjuvants (Fig 4A). The similar results were showed in the adjuvant H9 subtype inactivated vaccine. The HI antibody levels in chickens of H9-CVCVA5 vaccine group were significantly higher than those of H9 vaccine along group (Fig 4B).

### Supplementation of CVCVA5 adjuvant induces higher mucosal HI antibody titers and improves both Th1- and Th2-type cytokine levels

**Fig 5:**
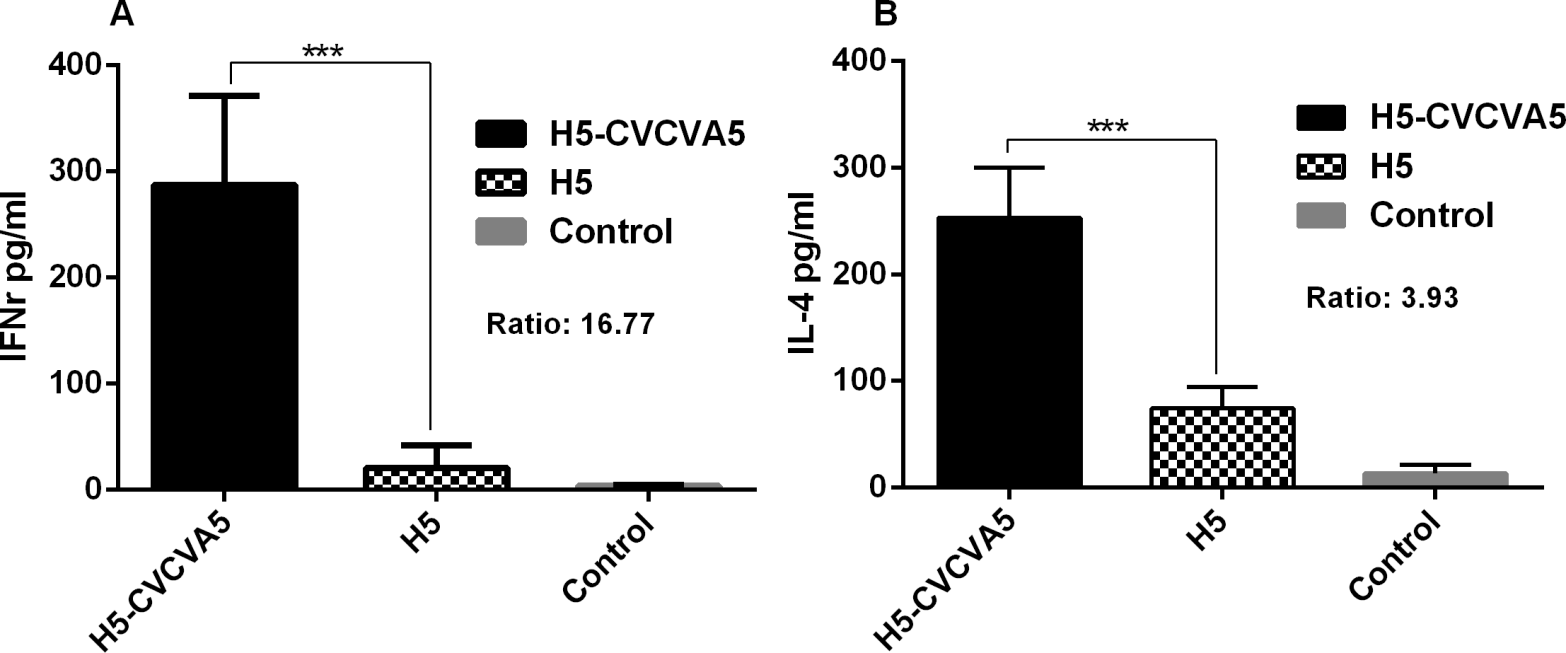
Serum cytokine levels from SPF chickens at 3 wpv. Levels of IFN-γ (A) and IL-4 (B) cytokines in SPF chickens serum were determined by ELISA Kit at three-week post vaccination from each group (n = 4). The values were represented mean±S.D. ***, *P* < 0.01. Ratio was counted as the following, (H5-CVCVA5-control)/ (H5-control).

We assayed the Th1-type cytokine levels of IFN-γ and Th2-type cytokine levels of IL-4 to evaluate the influence of CVCVA5 adjuvant on H5 vaccine in SPF chickens. Both cytokine levels of IFN-γ (Fig 5A) and IL-4 (Fig 5B) from serum of chickens vaccinated with adjuvant H5 vaccine were significantly improved when compared to the non-adjuvant H5 vaccine at 3 wpv. Similar results of these cytokine levels in serum were monitored in two batches of chickens vaccination experiments.

**Fig 6:**
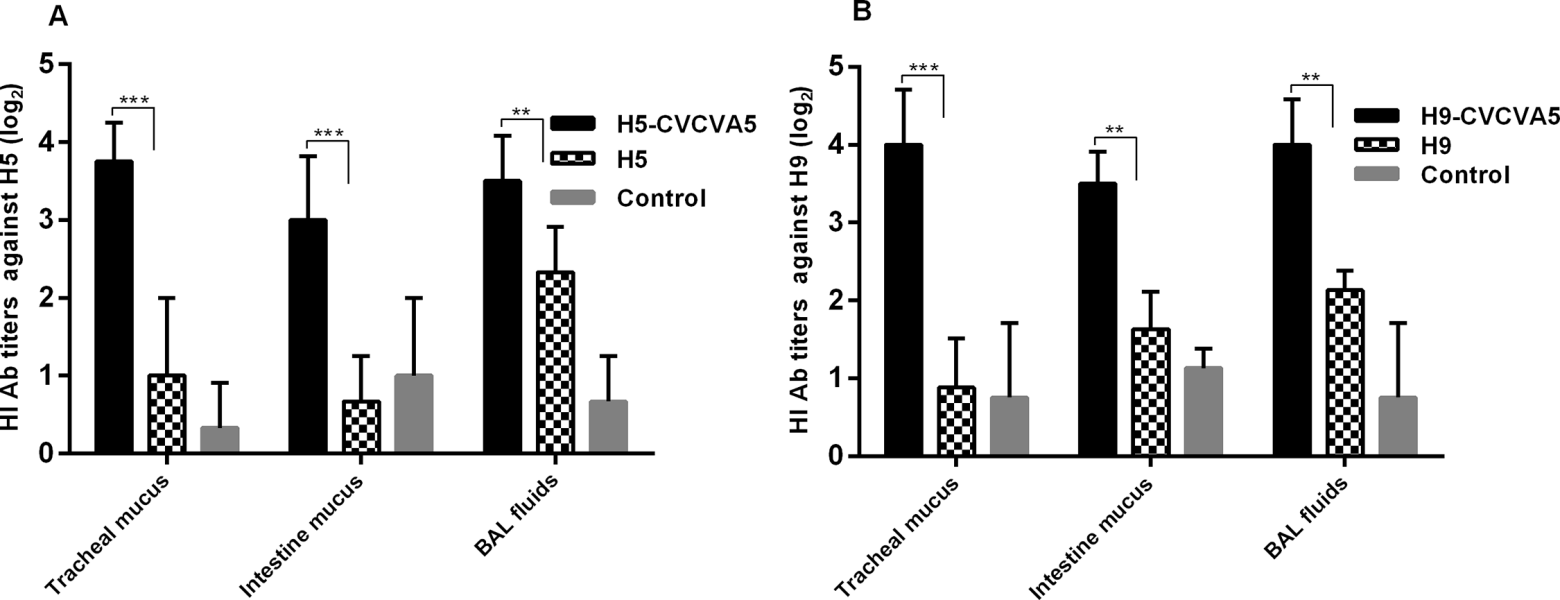
Mucosal antibodies against H5 or H9 subtype viral antigen at 3 wpv. SPF chickens were vaccinated with the adjuvanted H5-CVCVA5, H9-CVCVA5, H5 or H9 inactivated vaccine, respectively. The mucus was collected three-week post vaccination (n = 4) from each group. The mucosal antibody derived from scraps of trachea, small intestine and bronchoalveolar lavage fluids (BAL fluids) alone, against H5 (A) or H9 (B) viral antigen tested by HI assay, respectively. **, *P* < 0.05. ***, *P* < 0.01.

Mucosal antibody plays an important role in preventing AI virus natural infection [20]. To determine the efficacy of CVCVA5 adjuvants with H5 or H9 subtype vaccines on mucosal immune response in SPF chickens, mucus from trachea, small intestine and BAL fluids, were collected at 3 wpv for HI antibody measurement. Interestingly, the H5-CVCVA5 vaccinated group demonstrated a higher HI antibody levels than those of the commercial H5 vaccine group (Fig 6A). Similar discrepancies of H9 mucosal antibody levels were observed between those chickens injected with H9-CVCVA5 and H9 vaccine only (Fig 6B). The small intestine derived mucosal antibodies displayed nonspecific reaction to the H5 or H9 viral antigen.

### CVCVA5 adjuvant H5 vaccine elicits highly-neutralizing levels of cross-reactive antibodies

The neutralizing antibodies play a pivotal role in inhibiting and eradicating the infectious virus. The assay was test the cross neutralizing capability of antibodies derived from H5-CVCVA5 vaccine-immunized SPF chickens with the heterologous viruses from clade 7 and subclade 2.3.4.6 or homologous viruses from clade 2.3.4, respectively. Compared to the antibodies elicited by commercial H5 vaccine, the antibodies induced by H5-CVCVA5 vaccine showed stronger neutralization potency with both DT (subclade 7) and ZJ (subclade 2.3.4.6) viruses in 11-day old SPF chicken embryos (Table 2). Similar results were observed in the neutralization test with the homologous viruses S (subclade 2.3.4) strain.

**Table 2:** Serum neutralization test in 11-day old SPF chicken embryos.

Serum	Neutralization virus titer(EID_50_)		
	S (Clade 2.3.4)	DT(Clade7)	ZJ(Clade 2.3.4.6)
H5-CVCVA5	6.5	5.33	4.0
H5	5.42	4.5	2.8

SN assays were performed in 11-day old SPF chicken embryos with alpha method. A fixed serum was reacted with tenfold serial dilutions of virus solutions. The vaccine seed strain of H5 or H5-CVCVA5 were genetically located at clade 2.3.4. S virus was isolated from mallard and phylogenetically attributed to the same clade of vaccine seed strain. Both DT and ZJ viruses were isolated from chicken, and attributed to clade 7 or 2.3.4.6, respectively.

### The adjuvant vaccine improves the lymphocyte proliferation and CTL activity

**Fig 7:**
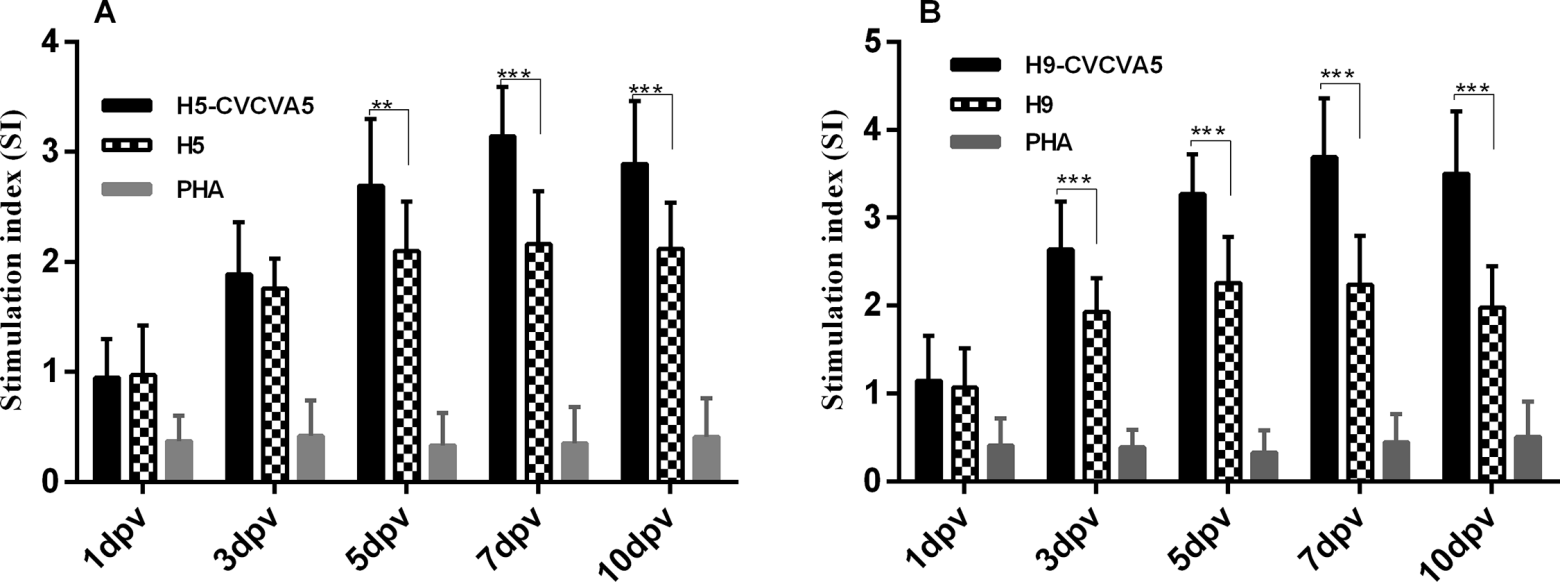
Mitogen-stimulated proliferation of splenocytes isolated from SPF chicken receiving H5 (Re-5) or H9 (NJ02/01) vaccine with or without CVCVA5 adjuvant. Splenocytes were prepared after the immunization and cultured with inactivated H5 (Re-5, 5μg/ml) or H9 (NJ02/01, 5μg/ml) viral antigen. Splenocytes proliferation was measured by the MTT method, and shown as a stimulation index. The values were represented mean ± S.D (n = 5) **, *P*<0.05. ***, *P*<0.01.

As shown in Fig 7A, increased SI magnitude of splenocytes proliferation from chickens vaccinated with the H5-CVCVA5 was observed in contrast to the non-adjuvant H5 vaccine group at day 3 post vaccination. From 5 to 10 days after immunization, SI of the chickens received injection with H5-CVCVA5 showed significantly increased when compared to those immunized with H5 vaccine alone, the latter likely to reach the plateau phase during the monitoring period. The adjuvant of CVCVA5 also play important role in stimulation of the lymphocyte proliferation in H9 vaccine injected chickens. The results of lymphocyte proliferation response in the non-adjuvant H9 vaccinated chickens were similar to those of chickens administered the H5 vaccine without adjuvant (Fig 7B).

**Fig 8:**
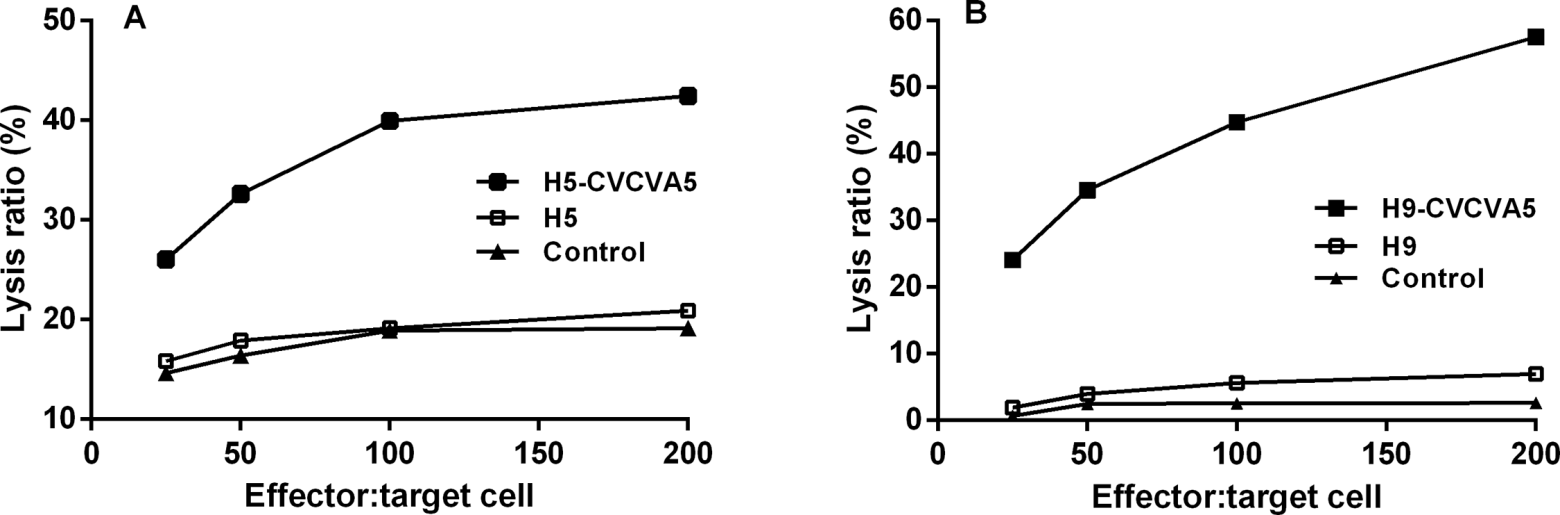
Cytotoxicity of CD8^+^ T cells from inbred chickens (B_19_/B_19_) vaccinated H5 (Re5) or H9 (NJ02/01) subtype vaccine with or without adjuvants. Inbred chicken embryo fibroblasts infected with S (H5N1) or NJ02/01 (H9N2) avian influenza viruses were used as target cells. PBMCs derived from the vaccinated inbred chicken or control group were used as effector cell.

The T cell provided protection against H9 subtype heterologous virus challenge in lymphocyte adoptive transfer assay in our previous study [15]. To further examine the mechanism of the T cell mediated protection, the CTL activity was detected. The effector cells of PBMCs from vaccinated chickens were incubated with H5 or H9 infected CEF as target cells at different ratios. The CTL activity of PBMCs from those birds vaccinated with the H5- or H9-CVCVA5 was dose related with the effector-to-target cell ratios. In contrast to the low lysis ratios of the chickens vaccinated with the H5 vaccine, the lysis effect of PBMCs from those birds vaccinated with the H5-CVCVA5 was sharply improved (Fig 8A). The CTL responses in the H5 vaccine immunized group were nearly at the same level to those of the unvaccinated control group. The adjuvant of CVCVA5 also significantly ameliorated the CTL response level in those chickens shot with H9-CVCVA5 vaccine in comparison with those of chickens received the H9 vaccine without adjuvant (Fig 8B). The CTL responses in the H9 vaccine immunized group were only slighter higher than those of the unvaccinated control group.

## Discussion

The inactivated H5 and H9 subtype AI vaccines play a vital role in helping to prevent and control of AI outbreaks and spread in China and other countries, in which existed numbers of village or backyard poultry raising farming [6, 8]. The serum antibody elicited by inactivated AI vaccine confers protection against homologous virus infections in the field. However, the antibody level is insufficient to limit the spread of viral variants [21]. Hence, a more comprehensive and efficient protection, including high cellular immune response and mucosal antibody are urgently needed in field use. The CVCVA5 adjuvants which we developed can improve both the serum antibody and cell mediated immune response [15].

A two-dose regimen of inactivated H9N2 AI vaccine is needed to enhance the immunologic response in broiler chickens [22]. Single vaccination does not reduce transmission of H5N1 highly pathogenic avian influenza viruses in commercial broilers [23]. However, the broiler chickens have a shorter life span than the laying hens, which requires a more rapid and efficient immune response after the vaccination to shorten the immunization blank period and quickly reach to the protective threshold levels of antibody. Significantly improved serum HI antibodies in broiler chickens with H5 AI vaccine containing adjuvants of CVCVA5 compared to those in chickens immunized with H5 AI vaccine only. The performances of production were scarcely influenced by the adjuvants based on the indexes of slaughter weights, food conversion ratio and death and culling rate. These data indicated a good application effect of the adjuvants on poultry production in commercial broilers.

Two-dose vaccination schedule is needed to elicit effective protection in ducks or geese according to the criteria of the commercial H5 AI vaccine or national recommended immunization program in the field application. The efficacy of different types of H5 vaccines are tested in the duck but not geese under laboratory settings in previous reports [24–26]. Comparison with emulsion formulations of commercial H5 inactivated vaccine, significantly improved the HI antibody levels were observed in both ducks and geese which shot with the CVCVA5-adjuvanted emulsion formulations inactivated H5 AI vaccine in this study.

Selection of appropriate vaccine strains raise many potential challenges and may result in suboptimal protection in field use [27]. Thus, improving the existing commercial vaccine efficacy to “universal” vaccines that induce cross neutralization antibody response against the diverse AIVs strains would eliminate much of the uncertainty associated with the vaccine candidate strains selection and impede emerging pandemic viruses. In this study, supplementation of the CVCVA5 adjuvant in the commercial H5 AI vaccine (clade 2.3.4) elicits high levels of cross neutralization antibody against the different phylogenic clades (clade 7) or subclades variant viruses (clade 2.3.4.6).

Without addition of the immunostimulatory components, most of the oil-based emulsion-inactivated AI vaccines induce overwhelming antibody responses and nearly undetectable cellular immune response [28]. The immune modulators, including agonists of toll-like receptor (TLR)-3 or -9 and chitosan, are efficacious in improving H5 AI oil-emulsion vaccine in chickens [29]. In this study, the integration of different immune modulators, including agonists of TLR-3, TLR-7/8, and others, into the compound adjuvants and mixing use with the H5 or H9 vaccine improved not only the humoral response but also the inflammation response which indexed by the increment of cytokine levels. Both Th1- (IFN-γ) and Th2-type (IL-4) cytokines were elevated. IFN-γ (ratio = 16.77) was more significantly expressed than IL-4 (ratio = 3.93) in terms of the ratio in adjuvant H5 vaccine group compared to the non-adjuvant H5 vaccine group, as shown in Fig 5. In contrast to the chickens received the non-adjuvant H5 or H9 vaccine group, the splenocyte proliferation in the H5- or H9-CVCVA5 vaccinated chickens were significantly benefit from the efficacy of the adjuvant CVCVA5 (Fig 7). The CTL activities in chickens of adjuvant H5 or H9 vaccine group were prominently higher than those of the H5 or H9 vaccine without adjuvant group (Fig 8), which consistent with the protection efficacy of adoptive CD8^+^ subtype lymphocytes transfer assay in our previous studies [15]. Combining the above descriptions, including the cytokine expression profiles, splenocyte proliferation and CTL response, indicated that the adjuvant CVCVA5 is an effective stimulator of cellular immune response in mixing use with H5 or H9 inactivated AI vaccine in the chickens.

The mucosal immune response is the first line of defense against influenza virus infection. However, the currently available parenteral influenza vaccine induces polarization of serum antibody immunity, which does not prevent influenza virus primary infection at the mucosal surface. The live-virus-vectored vaccines expressing the AI HA gene can stimulate mucosal immunity to AI viruses, such as the recombinant fowlpox or recombinant Newcastle disease based vector expressing the H5 or H7 HA. However, the maternal-derived anti-vector antibodies influenced the timing and route of application in field usage [28]. Addition of immune modulators can enhance the efficacy of mucosal immune response of inactivated AI vaccine [29]. The adjuvanted H9 AI vaccine reduces virus shedding after the H9 heterogeneous virus challenge in our previous study [15]. The mucosal HI antibodies level of scraps of trachea, small intestine and bronchoalveolar lavage fluids in chickens injection with H5 or H9 vaccine containing adjuvant markedly higher than those of H5 or H9 vaccine only. In most of the previous publications which associated with inducing mucosal antibody, the vaccine was delivered via intranasal or intradermal route. As we know, there is no report of administration of inactivated AI vaccine with or without adjuvants via subcutaneous route to elicit mucosal immune response. In this study, high levels of mucosal HI antibodies were induced by administration inactivated vaccine containing the adjuvant via subcutaneous route.

In summary, the present study demonstrated that the CVCVA5 adjuvant significantly improved the efficacy on commercial H5 and/or H9 subtype AI vaccines in comprehensive immune response, including serological and mucosal antibodies, cytokine and cellular responses in the chickens, ameliorated the HI antibody level in broiler chickens and ducks, prolonged immune persistence periods in geese. Therefore, the CVCVA5 based on the currently licensed AI vaccine has a great potential to become an effective adjuvant in poultry use.
